# Sailors and Defective Vision

**Published:** 1895-11-09

**Authors:** 


### Editor's Letter-Box.

[Our correspondents are reminded that prolixity is a great bar to publication, and that brevity of style and conciseness of statement greatly facilitate early insertion.]

### SAILORS AND DEFECTIVE VISION.

The Rev. Charles Hopkins, Abbey Camp, Alton, Hants, writes: I have read with great interest and satisfaction your outspoken article on the "eyesight examination system" for officers in our merchant service, as now conducted by the Board of Trade. I have been engaged in work amongst our merchant seamen for over ten years, and case after case bearing upon the hardships and injustice which you expose has come under my notice. Only last week a young fellow who has completed his apprenticeship wrote to tell me he had passed his examination satisfactorily in seamanship and navigation, but was disqualified on account of defective eyesight. The mother of this young fellow (a widow of very limited means) made a generous effort, and raised the necessary premium with which to apprentice her son in a good firm, looking forward, of course, to the time when he would advance in his profession and keep her in her old age. He must now leave the sea, or else remain as able seaman all his life at wages ranging from £2 10s. to £3 a month. Justice demands that the Board of Trade should require an examination in eyesight before a boy signs his indentures. The fact is many shipowners care little or nothing about the future of a lad so long as they can secure the premium, and make sure of being able to keep it. It matters little to them whether a lad runs away, or "gives up" the sea, or is disqualified from advancing in his profession, for he invariably has to sacrifice the premium already paid, and there are always plenty of others ready to come forward to take his vacant place, and repeat the same old game of chance. You have only to take up a morning paper to see how constantly such apprentices are advertised for. I have approached the Board of Trade successfully on other subjects, and if you will kindly furnish me with any evidence in your possession or place me in communication with those who can and will do so, I shall be only too glad to see if it is not possible to move the Board of Trade to make such examination of sight compulsory before a boy signs his indentures of apprenticeship.

